# Flexural Properties of Heat-Polymerized PMMA Denture Base Resins Reinforced with Fibers with Different Characteristics

**DOI:** 10.3390/polym15153211

**Published:** 2023-07-28

**Authors:** Kaan Yerliyurt, Taha Buğra Taşdelen, Özlem Eğri, Sinan Eğri

**Affiliations:** 1 Department of Prosthodontics, Faculty of Dentistry, Tokat Gaziosmanpaşa University, 60250 Tokat, Türkiye; 2 Institute of Graduate Studies, Bioengineering Division, Tokat Gaziosmanpaşa University, 60250 Tokat, Türkiye; 3 Department of Mechanical Engineering, Faculty of Engineering and Architecture, Tokat Gaziosmanpaşa University, 60250 Tokat, Türkiye; 4 Department of Chemistry, Faculty of Science and Letters, Tokat Gaziosmanpaşa University, 60250 Tokat, Türkiye

**Keywords:** denture base resin, glass fibers, polypropylene fibers, carbon fibers, reinforcement of polymethylmethacrylate

## Abstract

Polymethylmethacrylate (PMMA) has been the most-widely used denture base material in prosthetic dentistry for the last 80 years. It is still one of the best alternatives when new methods are inapplicable. Due to the lack of some physical inadequacies occurring during cyclic use and accidental situations, various reinforcement strategies such as using nanoparticles, wires, fibers, and meshes have been investigated and reported. In this study, it was aimed to conduct a comparative investigation of the effect of fiber additives with different characteristics on the flexural properties of heat-cured PMMA denture base resins. Glass fibers (GFs), polypropylene fibers (PPFs), and carbon fibers (CFs) having 3, 6, and 12 mm lengths and 0.25, 0.50, and 1.0% concentrations (*v*/*v*) were used for the reinforcement of PMMA denture base resins. The flexural properties (flexural strength, flexural modulus, and maximum deformation) were determined using a three-point bending test, and three-way ANOVA analyses with Bonferroni corrections were performed on the test results. The morphologies of the fracture surfaces were analyzed using scanning electron microscopy. All three fibers exhibited reinforcement in the flexural strength (*p* < 0.001) and flexural modulus (*p* < 0.001) regardless of their length and concentration. The group with 1.0% 12 mm CF-reinforced PMMA exhibited the greatest flexural strength (94.8 ± 8.8 MPa), and that with 1.0% 3 mm GFs displayed the lowest flexural strength (66.9 ± 10.4 MPa) among the fiber-reinforced groups. The greatest value of the flexural modulus was displayed by the 1.0% 3 mm CF-reinforced resin (3288.3 ± 402.1 MPa). Although the CF-reinforced groups exhibited better flexural properties, CFs are not favorable for use as reinforcement in practice due to the dark gray discoloration of the denture base resin. It was concluded that PPF is a promising material for the reinforcement of heat-cured PMMA denture base resins.

## 1. Introduction

Since the early 1940s, polymethylmethacrylate (PMMA) has become the most-widely preferred and used denture base material in prosthodontic dentistry because of its superior features such as its aesthetic appearance, being readily processable, and having a low cost [1,2,3,4,5]. Besides these, PMMA is biocompatible, safe, and dimensionally stable, has no taste or odor, and is non-irritating, non-toxic, stable in the oral environment, insoluble by saliva, and color-stable [6,7]. Despite these excellent properties, PMMA has unfavorable weaknesses in some of its mechanical and physical properties such as impact resistance, flexural strength, and fatigue fracture [8,9]. A great majority of dentures (63–68%) become useless because of fatigue fracture due to the chewing forces they are subjected to while in the mouth or impacts due to accidental dropping on hard surfaces while out of the mouth [10,11]. In order to avoid fractures, strategies such as reinforcing the PMMA denture base with metal wires have been tried, but poor adhesion between the metal wire surface and the PMMA matrix was the main problem with this strategy [9,11,12,13,14]. Another approach for reinforcement was forming a graft copolymer of PMMA and butadiene styrene, but it was found to be even weaker than PMMA because of the low bending strength compared to conventional acrylic resin, despite its high impact resistance [15,16].

Carbon fibers, aramid fibers, high-molecular-weight polyethylene fibers, and similar fibers have been used as denture base reinforcement materials, and it was reported that these fibers increased the bending and impact strength of the denture base resins [6,9,17,18,19]. Because of their biocompatibility, favorable aesthetics, and mechanical properties, nylon fibers, polyethylene fibers, polyamide fibers, and especially, glass fibers have been used in several studies [17,18,20,21]. The enhancing effect of glass fibers on the flexural strength and fatigue resistance of the PMMA denture base resin was previously reported [9,17,20,22,23]. Polypropylene (PP) was considered to be a suitable material for PMMA base resin reinforcement because of its prominent properties such as high-level resilience, elasticity, and tensile strength, durability in acids and similar mediums, low density (0.91 g/cm^3^), and low cost [24,25]. As an alternative, carbon fiber reinforcement was shown to increase the bending strength of the PMMA denture base resin [26].

In this study, it was aimed to comparatively investigate the effect of fiber reinforcement using fibers with different characteristics, namely made of different materials (glass, polypropylene, and carbon), and using three different fiber lengths (3, 6, and 12 mm) and three different concentrations (0.25, 0.50, and 1.0% *v*/*v*) on the mechanical properties of the PMMA denture base resin. As described above, various fibers were used to enhance the mechanical properties of the PMMA denture base resin, but none of these studies reported a comparative evaluation of the effects of the fiber materials, fiber lengths, or fiber concentrations on the flexural properties. In order to eliminate the effect of the different densities of each fiber material, volumetric ratios for the fiber reinforcement of the PMMA resin were used instead of weight ratios using appropriate calculations.

## 2. Materials and Methods

The heat-cured PMMA denture base resin was made of two components, powder and liquid (Akrodent, Koca Kimya ve Dental Ltd. Şti., Ankara, Turkey). The PP fibers (Polyfibers, İstanbul, Turkey), glass fibers (Dost Kimya, İstanbul, Turkey), and carbon fibers (Dost Kimya, İstanbul, Turkey) were used as received. No further treatment was performed on the fibers to maintain similar conditions for each fiber type. A two-piece mold was produced from chromium for the production of test samples for the control and fiber-reinforced groups with 65 × 10 × 3 mm^3^ dimensions to match the dimensions described in the ISO 178 standard [27].

There were three fiber-reinforced groups: glass fiber, PP fiber and carbon fiber, and each group was produced using 3, 6, and 12 mm fibers in 0.25, 0.50, and 1.0% *v*/*v* concentrations for each fiber type and length. A total of 224 samples in 28 groups (1 control and 27 with fiber reinforcement) and n = 8 samples for each group were formed for the three-point bending tests.

The ideal powder–liquid mixture ratio was used as 23.4 g powder to 10 mL liquid and complete wetting were observed. The acrylic paste was cast using a mold made of chrome designed with dimensions of 65 × 10 × 3 mm^3^. Then, the mold was pressed in the hydraulic press device (GLS, Gulersan Lubrication Equipment Industry and Trade Co., Ltd., Istanbul, Turkey) for 5 min, and the excess acrylic was removed. The reactions were carried out in a fully automatic polymerization device (MD-135, Meta Dental, Ankara, Turkey) by heating from room temperature (25 °C) to 90 °C and keeping the temperature constant for 20 min. At the end of the reaction period, the mold was taken out and then left to cool down to room temperature followed by the removal of the samples from the mold.

**Figure 1:**
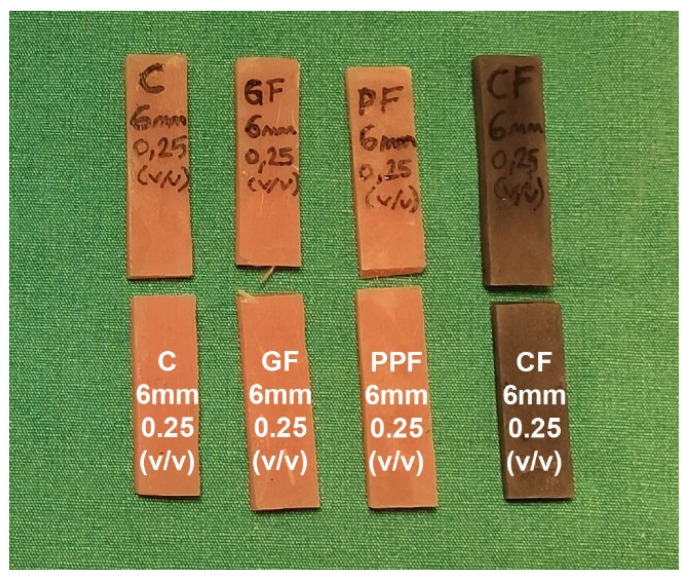
Test specimens (control, GF-, PPF-, and CF-reinforced) after three-point bending test.

The flexural properties of the control and fiber-reinforced groups were determined by the three-point bending tests by following the ISO 178 standard. A representative image of the test specimens after the three-point bending test is shown in Figure 1. The three-point bending tests were performed using a universal testing machine (Autograph AGS-X, Shimadzu, Kyoto, Japan) at a compression rate of 10 mm/min on the samples placed between the shoulders having a gap of 50 mm. All tests were carried out at room temperature, and the mechanical properties of the samples were investigated.

The morphologies of the fracture surfaces that were obtained from the three-point bending tests were investigated through the SEM images taken of their surfaces. Fractured test specimens were cut to reduce the height of the sample to 5 mm long for the SEM analyses. The test specimens were positioned on the sample tray with the fracture surfaces facing up. The SEM specimens were coated with Au under a vacuum atmosphere in a coating instrument (Quorum Q150RES, Birmingham, U.K.). The SEM images were collected by the SEM equipment (Tescan Mira3 XMU, Brno, Czechia) with an accelerating voltage of 10 kV.

The flexural strength, flexural modulus, and maximum deformation values from the three-point bending tests were analyzed using the three-way ANOVA test, and the level of significance was set to *p* < 0.05. Bonferroni corrections were performed for multiple comparisons. All the statistical analyses were conducted using the statistics software SPSS 20 (IBM, Chicago, IL, USA).

## 3. Results

### 3.1. Three-Point Bending Tests

All test specimens were subjected to a three-point bending test, and the collected data for 28 groups including the control and fiber-reinforced groups (n = 8 samples in each group) were tested with three-way ANOVA using Bonferroni corrections. Statistical analyses were conducted for three flexural parameters from the three-point bending test, flexural strength (MPa), maximum deformation (mm), and flexural modulus (MPa). Multiple comparisons were performed to determine the effect of the fiber material (material), fiber-volume-to-resin-volume (concentration), and fiber length (length) on the flexural properties. According to the statistical analyses, any type of fiber material (GF, PPF, and CF) regardless of the fiber length and concentration used for denture base resin reinforcement exhibited a significant change in all three flexural parameters (*p* < 0.001). The concentration of the fiber used exhibited a significant difference only in the maximum deformation (*p* < 0.001). Statistical analyses revealed that the fiber concentration (*p* = 0.273) or fiber length (*p* = 0.211) had no significant effect on the flexural strength regardless of the fiber material used.

**Figure 2:**
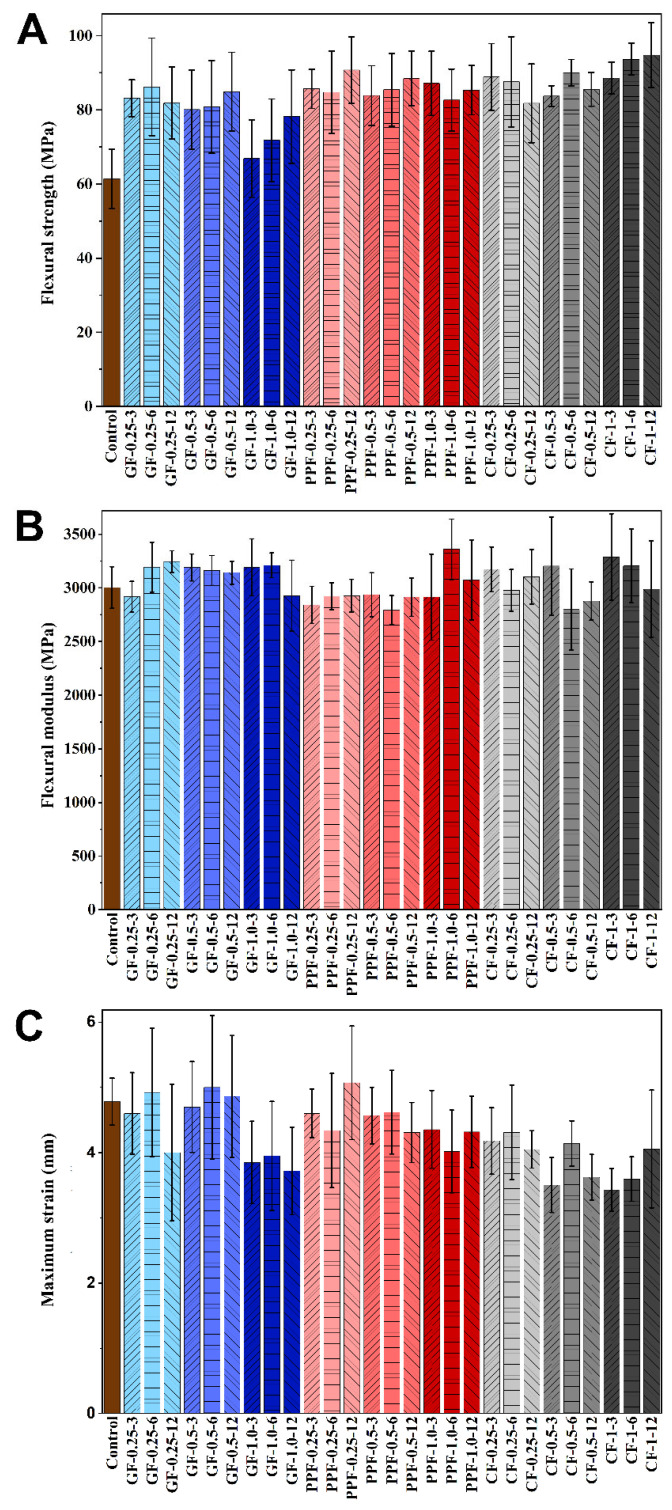
Flexural properties of the test groups obtained by three-point bending test; (**A**) flexural strength (MPa); (**B**) flexural modulus (MPa); (**C**) maximum deformation (mm).

The means with the standard deviations and scoring on the significance of the tested variables obtained by the comparisons of the three-point bending test results are presented in Table 1 for the flexural strength, Table 2 for the flexural modulus, and Table 3 for the maximum deformation. Furthermore, the test results obtained by the three-point bending tests are plotted and presented in Figure 2.

The PPFs and CFs used for reinforcement exhibited a significant increase in flexural strength compared to reinforcement with GFs (*p* < 0.001). Although the concentration did not result in a significant change for the PPF reinforcement, the use of GFs at 1.0% ratio resulted in a decrease and CFs at 1.0% resulted in an increase in flexural strength regardless of the GF and CF length (*p* < 0.001). The greatest value of the flexural strength was observed as 94.8 ± 8.8 MPa with 1.0% 12 mm CFs, and the lowest was 66.9 ± 10.4 MPa for the 1.0% 3 mm GF-reinforced PMMA denture base resin. The concentration, as well as the length did not reveal a significant change in flexural strength compared to each fiber-reinforced group, but a significant change was observed in comparison with the control group (*p* < 0.001). Comparing the concentration and material regardless of length, the 0.25 and 0.50% fiber-reinforced denture base resins did not exhibit a significant change, while 1.0% exhibited a significant increase when comparing GFs with PPFs (*p* < 0.001) and PPFs with CFs (*p* < 0.05). Comparing the effect of the concentration in each material group, the 1.0% GF-reinforced resin showed significantly lower flexural strength than the 0.25 and 0.50% GF-reinforced groups (*p* < 0.001); contrary to GFs, the 1.0% CF reinforcement resulted in a significant increase compared to the 0.25% CF-reinforced group, and the PPF reinforced group did not exhibit a significant difference with the concentration (*p >* 0.05) (Table 1 and Figure 2A).

Since the length did not show any significant difference in each material group regardless of the concentration, 3 mm PPF- (*p* < 0.05) and 3 mm CF- (*p* < 0.001) reinforced groups resulted in higher flexural strength values, while 6 mm CF reinforcement increased it significantly (*p* < 0.001), and 6 mm PPF group increased it comparably (*p* = 0.056) in comparison to the 6 mm GF reinforcement group. The flexural strength values of the 12 mm PPF were significantly higher (*p* < 0.05), and the 12 mm CFs were comparable (*p* = 0.086) with the 12 mm GF reinforcement. The concentration vs. length did not exhibit a significant change in the flexural strength in comparison regardless of the material (*p >* 0.05) (Table 1).

Like the flexural strength, all three parameters, material, concentration, and length, used for fiber reinforcement resulted in a significant increase in the flexural modulus compared to the control (*p* < 0.001). However, this difference was significantly higher for CF reinforcement (*p* < 0.001), and the difference between GF and PPF reinforcement was insignificant (*p >* 0.05). Both the concentration and length did not exhibit significant differences for any of the materials used for reinforcement (*p* = 1.000). The greatest value of the flexural modulus was observed as 3288.3 ± 402.1 MPa with 1.0% 3 mm CFs, and the lowest was 2495.4 ± 324.1 MPa for the 1.0% 3 mm GF-reinforced PMMA denture base resin. Comparing the material and concentration regardless of the length, the 0.25 and 0.50% fiber-reinforced denture base resins did not exhibit a significant change, while the 1.0% CF-reinforced group exhibited a significant increase compared to the GF-reinforced group (*p* < 0.001) and PPF-reinforced group (*p* < 0.001). The length did not show any significant difference in each material group regardless of the concentration; the 3 mm CF-reinforced group exhibited a significantly higher flexural modulus than the GF and PPF groups (*p* < 0.001), and the flexural modulus of the CF group decreased by increasing the length to 12 mm (*p* < 0.001) (Table 2 and Figure 2B).

Comparing the maximum deformation for the material and concentration, only the CF reinforcement exhibited a significant decrease (*p* < 0.05), while the GF and PPF reinforcement did not show a significant change (*p >* 0.05). Using PPFs as reinforcement did not significantly change the maximum deformation at any concentration and length (*p >* 0.05). The highest was observed to be 5.1 ± 0.9 mm for the PPF-reinforced group with a 12 mm length and a 0.25% concentration (Table 3 and Figure 2C).

### 3.2. SEM Analysis

**Figure 3:**
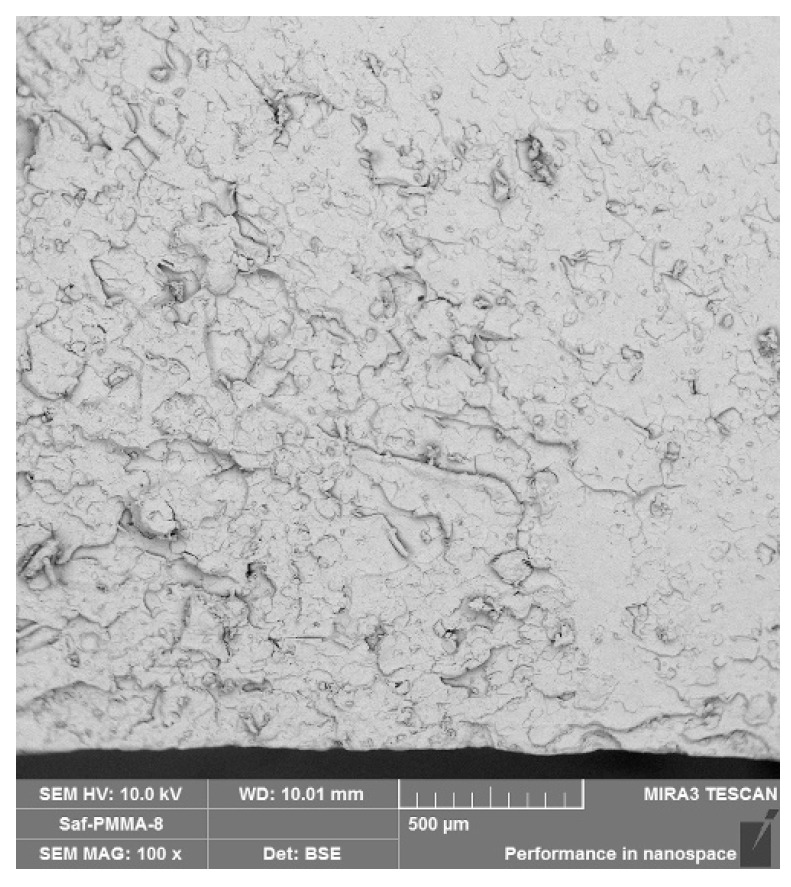
The 100× SEM image taken from the fracture surface of the control group.

**Figure 4:**
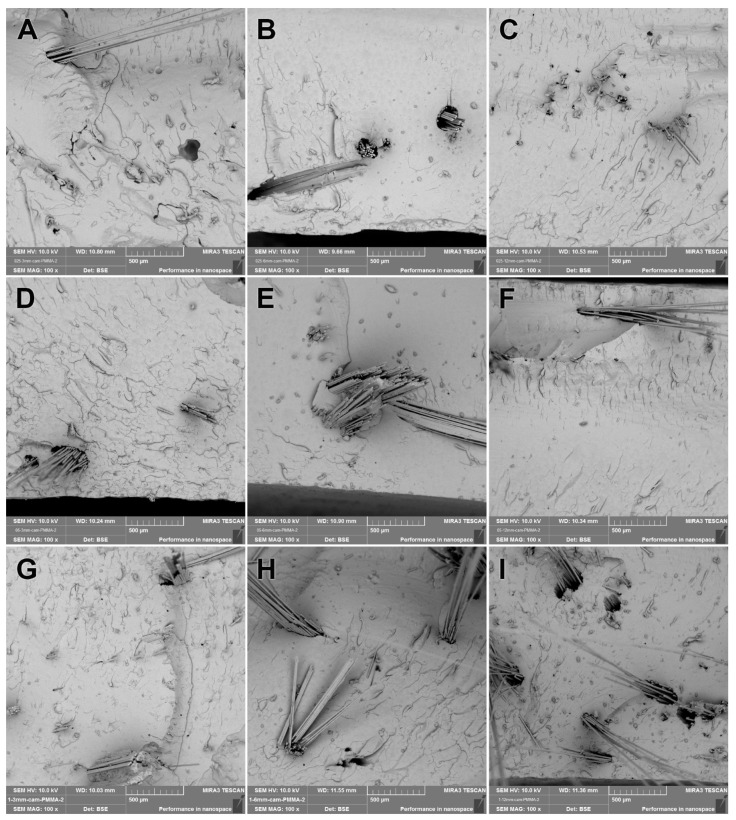
The 100× SEM image taken from the fracture surface of the GF group. (**A**) GF-0.25-3; (**B**) GF-0.25-6; (**C**) GF-0.25-12; (**D**) GF-0.50-3; (**E**) GF-0.50-6; (**F**) GF-0.50-12; (**G**) GF-1.0-3; (**H**) GF-1.0-6; (**I**) GF-1.0-12.

**Figure 5:**
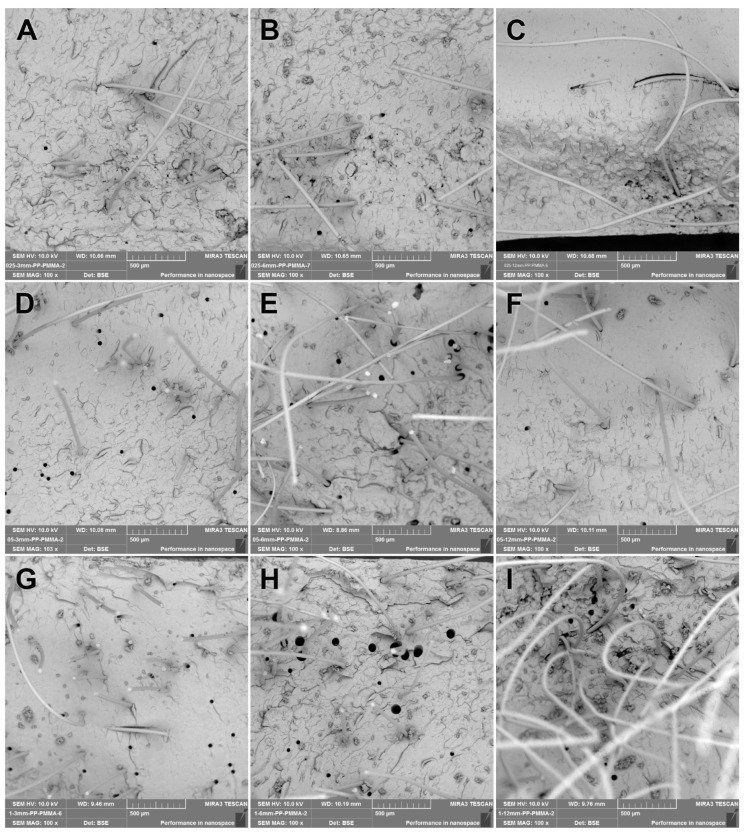
The 100× SEM image taken from the fracture surface of the PPF group. (**A**) PPF-0.25-3; (**B**) PPF-0.25-6; (**C**) PPF-0.25-12; (**D**) PPF-0.50-3; (**E**) PPF-0.50-6; (**F**) PPF-0.50-12; (**G**) PPF-1.0-3; (**H**) PPF-1.0-6; (**I**) PPF-1.0-12.

**Figure 6:**
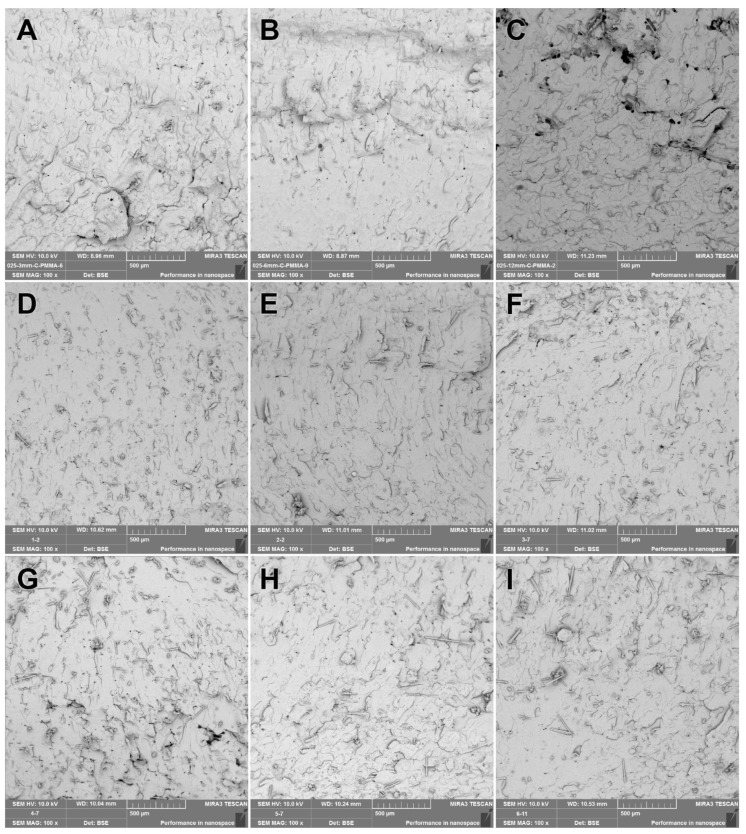
The 100× SEM image taken from the fracture surface of the CF group. (**A**) CF-0.25-3; (**B**) CF-0.25-6; (**C**) CF-0.25-12; (**D**) CF-0.50-3; (**E**) CF-0.50-6; (**F**) CF-0.50-12; (**G**) CF-1.0-3; (**H**) CF-1.0-6; (**I**) CF-1.0-12.

SEM images were obtained from the fracture surfaces of the specimens used in the three-point bending tests. The 100× SEM images from the fractured control, GF-reinforced denture base resin, PPF-reinforced denture base resin, and CF-reinforced denture base resin materials are presented in Figure 3, Figure 4, Figure 5, and Figure 6, respectively. The SEM image analyses of the control group revealed that the base resin exhibited a brittle fracture under three-point bending test conditions.

It was seen from the SEM images of the GF-reinforced denture base resins that GFs had not been distributed homogeneously along the section of the specimen. The PMMA matrix residues observed on the broken GFs’ surfaces indicated that adhesive attachment between the PMMA matrix and the GF surfaces occurred, and fiber–matrix integration resulted in a better strength under compressive load. The aggregation of the GFs within the PMMA matrix was reduced by increasing the GF length. The denture base resins reinforced with 3 mm and 6 mm GFs exhibited relatively higher local fiber aggregation than the 12 mm GFs (Figure 4A–C). The higher concentrations of GFs used resulted in denser GF clusters within the matrix (Figure 4B,E,H).

The SEM images revealed that all PPF-reinforced groups showed a better fiber distribution compared to all GF-reinforced groups (Figure 5). Better interfacial matching (closeness of fitting) was observed for all PPF-reinforced groups, but weaker interfacial adhesion resulted in stripped PPFs from the opposite fracture pieces of the test specimen (Figure 5D,E). A ductile fracture was observed for longer PPFs used for reinforcement. Holes were seen on the SEM images (i.e., Figure 5H) formed by stripping of the PPFs due to load application during three-point bending test. The gap between the PPFs and the matrix (Figure 5E) was formed by the plastic deformation of PPFs due to load application during the three-point bending test. Due to the random orientation of the PPFs due to the dense and longer fibers used, they distributed along all directions and, therefore, enhanced the flexural strength (Figure 5I).

An almost perfect fiber distribution was present for the CF-reinforced groups (Figure 6). This resulted in a perfect fracture interface. Increasing the fiber lengths resulted in holes across the fracture surface, but not as much as the PPF-reinforced groups (Figure 6B). CFs were better distributed along the PMMA matrix and resulted in better interfacial interaction than both the GF and PPF groups.

## 4. Discussion

In this study, glass fiber, polypropylene fiber, and carbon fiber groups were set and added to a heat-polymerized denture base resin with fiber-to-resin-volume ratios of 0.25, 0.50, and 1.0% using 3, 6, and 12 mm-long fibers for each group, and their effects on reinforcing of the heat-cured PMMA denture base resins were evaluated. The flexural strength, flexural modulus, and maximum deformation by three-point bending tests were compared in order to determine the reinforcing effect. All of the test specimens were kept in distilled water for two months, after fabrication prior to testing according to the storage periods reported [5,28,29,30,31,32,33]. It was reported that a reduction in flexural strength occurs in the first four weeks of immersion [34,35].

Reinforcement using GF significantly enhanced the flexural strength compared to the control group according to the three-point bending test results. This situation is parallel to the studies reported by Al-Thobity and Singh et al., in which the flexural strength of the PMMA denture base resins was improved with GFs [17,32]. Yu et al. also reported the enhancement of the flexural strength by reinforcement with GFs, but they used a local placement and orientation of the fibers [36]. The use of GFs with a 1.0% fiber-to-resin-volume ratio for all fiber lengths resulted in a decrease in the flexural strength, which can be explained by the increase in the void space between fiber clusters formed by the agglomeration of poorly distributed GFs. These void spaces between fibers caused the discontinuity of the resin matrix and the formation of weaker spots for the enhancement of crack propagation during fracture. Similar to the reinforcement with GFs, the reinforcement using PPFs and CFs significantly enhanced the flexural strength compared to the control group. However, the reinforcement using PPFs and CFs exhibited higher flexural strength regardless of the fiber length and concentration compared to the reinforcement with GFs. The concentration of the fiber used did not cause a significant change in the flexural strength for PPF reinforcement with any fiber length. Although no significance was obtained for the PP fiber length and concentration on the flexural strength, a slight increase in the flexural strength for the reinforcement with short PPFs by increasing the concentration and a slight decrease in the flexural strength for the reinforcement with long PPFs by increasing the concentration were observed, as was reported by Mathew et al. [37]. It was observed during the three-point bending tests that the maximum deformation of the resin samples decreased by increasing the fiber concentrations used for reinforcement. Consequently, it can be concluded that PMMA matrices reinforced with a high concentration of PPFs fractured with lower deflection due to a lack of maintaining the interfacial stability by distributing the compressive load appropriately on the fibers. Ismaeel et al. used PPFs that were surface modified with plasma application in order to enhance the mechanical properties of the PMMA denture base resin by increasing the fiber matrix interfacial adhesion [38]. The CF-reinforced resin exhibited high flexural strength values compared to the control. Similarly, Ma and Chen reported the greatest flexural strength by CFs in their study, where they compared GFs, CFs, and Kevlar fibers on the mechanical and thermal properties of PMMA composites [39]. Although better flexural strength values were obtained for resins reinforced using CFs, it is not possible to use these fibers for the reinforcement of denture base resins for clinical use because of the dark grey color they provide to the denture base.

The flexural modulus was observed to be greater for CF-reinforced resins at a 1.0% concentration and a 3 mm fiber length. This behavior may be attributed to the better interfacial match and possible physical and/or chemical bonding between the CF surface and PMMA matrix, which was assisted by shorter fibers homogeneously distributed along the matrix. As a result of this, a better distribution of the load between the matrix and fibers led the test specimen to resist higher compressive forces before fracture.

Comparing the maximum deformations of the GF-, PPF-, and CF-reinforced denture base resins, the CF-reinforced resins exhibited a lower deflection limit than the GF- and PPF-reinforced resins due to the less-ductile character provided by the relatively shorter fibers, which maintained a better interfacial match between the fibers and the matrix.

The SEM analyses of the GF-reinforced groups revealed that the distribution of GFs along the PMMA matrix was not good, even though they were observed to enhance the flexural strength. Therefore, processing of the GF surface modification may enhance the interfacial match of the GFs with the PMMA matrix by physical/chemical bonding. The PPFs were seen to be better distributed in the PMMA matrix, but adhesion between the PPFs and the matrix was weak, so that the fibers at the fracture plane were stripped from the opposite specimen parts by the load application. If the adhesion between the fibers and the PMMA matrix were enhanced, the mechanical properties could also be enhanced. PPFs are a good reinforcing material for denture base resins since they increase the flexural properties and are also non-toxic to the biological environment [40]. The CFs were observed to be homogenously distributed within the PMMA matrix. Therefore, CF-reinforced denture base resins were found to have the highest flexural strength.

It was aimed in this study to compare the effect of three different parameters on three flexural properties of heat-cured PMMA denture base resins, and promising enhancement of the flexural properties with any type of fiber material was observed. The main limitation of this study was the lack of the enhancement of pristine fiber–matrix adhesion. There are strategies such as the surface modification of the fibers used in order to enhance the adhesion, i.e., silanization of GFs resulting in a better dispersion and adhesion within the PMMA matrix [29]. Similarly, PPFs can also be modified, and the surface properties together with the fiber–matrix adhesion can be enhanced [38]. However, these processes are dependent on the modification parameters, which would indirectly affect the flexural properties. Pristine forms of the fibers were used in this study to avoid the insertion of direct and indirect variables into the experimental setup, which would not be comparable because of the different physical/chemical processes used for the modification of different fiber materials. CF reinforcement is not an option for the reinforcement of PMMA denture bases due to the dark color provided, but CFs were used in this study to emphasize the comparable enhancement in the flexural properties provided by PPF reinforcement.

## 5. Conclusions

All tests and analyses performed within the context of this study revealed that all fiber types can enhance the mechanical properties such as the flexural strength, flexural modulus, and maximum deformation. All three fibers exhibited reinforcement in the flexural strength regardless of their length and concentration. Although the fiber–resin interaction was observed to be poor, the reinforcement with PPFs provided quite good mechanical properties comparable to the reinforcement using GFs or CFs. Therefore, the PPF-reinforced denture base resins were concluded to be promising materials when cyclic loads such as chewing, etc., in the mouth are considered. Enhanced interfacial adhesion and the ductile character of surface-modified/-treated PPFs may reveal outstanding results for the reinforcement of heat-cured PMMA denture base resins.

## Figures and Tables

**Table 1:** Means, standard deviations, and significance by pairwise comparisons for flexural strength.

Comparison		Flexural Strength (MPa)			
Material		Control	Glass Fiber	PP Fiber	Carbon Fiber
		61.4 ± 8.0 ^(o)^	79.3 ± 11.9 ^(n)^	86.0 ± 8.3 ^(m)^	88.3 ± 8.1 ^(m)^
Concentration		0%	0.25%	0.50%	1.0%
		61.4 ± 8.0 ^(b)^	85.6 ± 9.7 ^(a)^	84.8 ± 8.5 ^(a)^	83.2 ± 12.2 ^(a)^
Length		0 mm	3 mm	6 mm	12 mm
		61.4 ± 8.0 ^(y)^	83.1 ± 9.6 ^(x)^	84.8 ± 11.2 ^(x)^	85.7 ± 9.9 ^(x)^
Material × Concentration			Glass Fiber	PP Fiber	Carbon Fiber
		0.25%	83.8 ± 9.6 ^(m,a)^	87.0 ± 8.8 ^(m,a)^	86.0 ± 10.7 ^(m,b)^
		0.50%	81.9 ± 11.0 ^(m,a)^	85.9 ± 8.3 ^(m,a)^	86.4 ± 4.5 ^(m,ab)^
		1.0%	72.3 ± 11.9 ^(o,b)^	85.0 ± 7.8 ^(n,a)^	92.3 ± 6.5 ^(m,a)^
Material × Length			Glass Fiber	PP Fiber	Carbon Fiber
		3 mm	76.7 ± 11.3 ^(n,x)^	85,6 ± 7,3 ^(m,x)^	87,0 ± 6,2 ^(m,x)^
		6 mm	79,6 ± 13,2 ^(n,x)^	84,3 ± 9,5 ^(mn,x)^	90,4 ± 7,9 ^(m,x)^
		12 mm	81,6 ± 10,9 ^(n,x)^	88,2 ± 7,7 ^(m,x)^	87,4 ± 9,8 ^(mn,x)^
Concentration × Length			3 mm	6 mm	12 mm
		0.25%	85.9 ± 6.8 ^(a,x)^	86.2 ± 11.7 ^(a,x)^	84.8 ± 10.3 ^(a,x)^
		0.50%	82.6 ± 7.7 ^(a,x)^	85.4 ± 9.8 ^(a,x)^	86.3 ± 7.7 ^(a,x)^
		1.0%	80.9 ± 12.8 ^(a,x)^	82.7 ± 12.2 ^(a,x)^	86.1 ± 11.5 ^(a,x)^
Material × Concentration × Length			Glass Fiber	PP Fiber	Carbon Fiber
	3 mm	0.25%	83.1 ± 5.0 ^(m,a,x)^	85.7 ± 5.3 ^(m,a,x)^	88.9 ± 9.0 ^(m,a,x)^
		0.50%	80.1 ± 10.7 ^(m,a,x)^	83.9 ± 8.0 ^(m,a,x)^	83.7 ± 2.8 ^(m,a,x)^
		1.0%	66.9 ± 10.4 ^(n,b,y)^	87.1 ± 8.7 ^(m,a,x)^	88.6 ± 4.3 ^(m,a,x)^
	6 mm	0.25%	86.2 ± 13.2 ^(m,a,x)^	84.8 ± 11.2 ^(m,a,x)^	87.6 ± 12.2 ^(m,a,x)^
		0.50%	80.8 ± 12.5 ^(m,ab,x)^	85.4 ± 9.8 ^(m,a,x)^	90.0 ± 3.6 ^(m,a,x)^
		1.0%	71.8 ± 11.2 ^(n,b,xy)^	82.6 ± 8.3 ^(n,a,x)^	93.7 ± 4.3 ^(m,a,x)^
		0.25%	81.8 ± 9.7 ^(m,a,x)^	90.7 ± 9.0 ^(m,a,x)^	81.8 ± 10.6 ^(m,b,x)^
	12 mm	0.50%	84.9 ± 10.7 ^(m,a,x)^	88.5 ± 7.4 ^(m,a,x)^	85.6 ± 4.6 ^(m,ab,x)^
		1.0%	78.2 ± 12.6 ^(n,a,x)^	85.3 ± 6.7 ^(mn,a,x)^	94.8 ± 8.8 ^(m,a,x)^

Three-way variance analysis was used, comparisons; (xy): for fiber length (ab): for concentration, (mno): for fiber material; values with different superscript letters indicate a significant difference (the first comparisons of triple parameters are colored in the same color to emphasize the values to be compared).

**Table 2:** Means, standard deviations, and significance by pairwise comparisons for flexural modulus from three-point bending test results.

Comparison		Flexural Modulus (MPa)			
Material		Control	Glass Fiber	PP Fiber	Carbon Fiber
		1924.1 ± 199.7 ^(o)^	2738.9 ± 243.9 ^(n)^	2829.1 ± 179.0 ^(n)^	3004.3 ± 365.2 ^(m)^
Concentration		0%	0.25%	0.50%	1.0%
		1924.1 ± 199.7 ^(b)^	2829.6 ± 206.6 ^(a)^	2864.8 ± 276.4 ^(a)^	2877.8 ± 376.1 ^(a)^
Length		0 mm	3 mm	6 mm	12 mm
		1924.1 ± 199.7 ^(y)^	2869.8 ± 354.3 ^(x)^	2826.4 ± 271.3 ^(x)^	2876.0 ± 247.4 ^(x)^
Material × Concentration			Glass Fiber	PP Fiber	Carbon Fiber
		0.25%	2743.6 ± 183.9 ^(m,a)^	2852.5 ± 164.4 ^(m,a)^	2892.7 ± 241.9 ^(m,b)^
		0.50%	2784.7 ± 204.1 ^(m,a)^	2850.1 ± 169.5 ^(m,a)^	2959.6 ± 386.2 ^(m,b)^
		1.0%	2688.2 ± 321.2 ^(n,a)^	2784.7 ± 200.1 ^(n,a)^	3160.6 ± 404.2 ^(m,a)^
Material × Length			Glass Fiber	PP Fiber	Carbon Fiber
		3 mm	2676.5 ± 259.8 ^(n,x)^	2810.2 ± 202.7 ^(n,x)^	3122.8 ± 411.7 ^(m,x)^
		6 mm	2715.9 ± 212.0 ^(n,x)^	2805.5 ± 180.2 ^(mn,x)^	2957.8 ± 344.4 ^(m,xy)^
		12 mm	2824.1 ± 242.8 ^(m,x)^	2871.6 ± 150.2 ^(m,x)^	2932.3 ± 318.3 ^(m,y)^
Concentration × Length			3 mm	6 mm	12 mm
		0.25%	2823.4 ± 220.7 ^(a,x)^	2783.0 ± 133.8 ^(a,x)^	2882.3 ± 245.0 ^(a,x)^
		0.50%	2932.7 ± 338.4 ^(a,x)^	2778.8 ± 272.5 ^(a,x)^	2882.9 ± 185.0 ^(a,x)^
		1.0%	2853.3 ± 466.5 ^(a,x)^	2917.4 ± 349.4 ^(a,x)^	2862.8 ± 307.2 ^(a,x)^
Material × Concentration × Length			Glass Fiber	PP Fiber	Carbon Fiber
	3 mm	0.25%	2732.6 ± 220.0 ^(m,a,x)^	2860.9 ± 141.6 ^(m,a,x)^	2876.8 ± 277.9 ^(m,b,x)^
		0.50%	2801.5 ± 96.8 ^(n,a,x)^	2793.4 ± 177.1 ^(n,a,x)^	3203.3 ± 458.3 ^(m,a,x)^
		1.0%	2495.4 ± 324.1 ^(n,a,y)^	2776.3 ± 281.1 ^(n,a,x)^	3288.3 ± 402.1 ^(m,a,x)^
	6 mm	0.25%	2739.2 ± 103.4 ^(m,a,x)^	2740.3 ± 121.1 ^(m,a,x)^	2869.6 ± 143.5 ^(m,b,x)^
		0.50%	2679.3 ± 171.7 ^(m,a,x)^	2858.5 ± 229.3 ^(m,a,x)^	2798.6 ± 378.1 ^(m,b,y)^
		1.0%	2729.1 ± 324.4 ^(n,a,xy)^	2817.8 ± 176.6 ^(n,a,x)^	3205.3 ± 343.8 ^(m,a,x)^
		0.25%	2758.9 ± 227.2 ^(m,a,x)^	2956.4 ± 165.3 ^(m,a,x)^	2931.7 ± 303.1 ^(m,a,x)^
	12 mm	0.50%	2873.4 ± 275.8 ^(m,a,x)^	2898.4 ± 64.0 ^(m,a,x)^	2876.9 ± 178.5 ^(m,a,y)^
		1.0%	2840.2 ± 241.0 ^(m,a,x)^	2760.0 ± 139.2 ^(m,a,x)^	2988.2 ± 449.5 ^(m,a,x)^

Three-way variance analysis was used, comparisons; (xy): for fiber length (ab): for concentration, (mno): for fiber material; values with different superscript letters indicate a significant difference (the first comparisons of triple parameters are colored in same color to emphasize the values to be compared).

**Table 3:** Means, standard deviations, and significance by pairwise comparisons for maximum deformation from three-point bending test results.

Comparison		Maximum Deformation (mm)			
Material		Control	Glass Fiber	PP Fiber	Carbon Fiber
		4.8 ± 0.4 ^(m)^	4.4 ± 1.0 ^(m)^	4.5 ± 0.7 ^(m)^	3.9 ± 0.6 ^(n)^
Concentration		0%	0.25%	0.50%	1.0%
		4.8 ± 0.4 ^(a)^	4.5 ± 0.8 ^(a)^	4.4 ± 0.8 ^(a)^	3.9 ± 0.7 ^(b)^
Length		0 mm	3 mm	6 mm	12 mm
		4.8 ± 0.4 ^(x)^	4.2 ± 0.7 ^(x)^	4.3 ± 0.9 ^(x)^	4.2 ± 0.9 ^(x)^
Material × Concentration			Glass Fiber	PP Fiber	Carbon Fiber
		0.25%	4.5 ± 1.0 ^(m,a)^	4.7 ± 0.8 ^(m,a)^	4.2 ± 0.6 ^(m,a)^
		0.50%	4.9 ± 1.0 ^(m,a)^	4.5 ± 0.6 ^(m,a)^	3.8 ± 0.5 ^(n,a)^
		1.0%	3.8 ± 0.7 ^(mn,b)^	4.2 ± 0.6 ^(m,a)^	3.7 ± 0.6 ^(n,a)^
Material × Length			Glass Fiber	PP Fiber	Carbon Fiber
		3 mm	4.4 ± 0.8 ^(m,x)^	4.5 ± 0.5 ^(m,x)^	3.7 ± 0.5 ^(n,x)^
		6 mm	4.6 ± 1.1 ^(m,x)^	4.3 ± 0.8 ^(mn,x)^	4.0 ± 0.6 ^(n,x)^
		12 mm	4.2 ± 1.0 ^(mn,x)^	4.6 ± 0.8 ^(m,x)^	3.9 ± 0.6 ^(n,x)^
Concentration × Length			3 mm	6 mm	12 mm
		0.25%	4.5 ± 0.6 ^(a,x)^	4.5 ± 0.9 ^(a,x)^	4.4 ± 1.0 ^(a,x)^
		0.50%	4.3 ± 0.8 ^(ab,x)^	4.6 ± 0.9 ^(a,x)^	4.3 ± 0.8 ^(a,x)^
		1.0%	3.9 ± 0.7 ^(b,x)^	3.9 ± 0.7 ^(b,x)^	4.0 ± 0.8 ^(a,x)^
Material × Concentration × Length			Glass Fiber	PP Fiber	Carbon Fiber
	3 mm	0.25%	4.6 ± 0.7 ^(m,a,xy)^	4.6 ± 0.4 ^(m,a,x)^	4.2 ± 0.6 ^(m,a,x)^
		0.50%	4.7 ± 0.7 ^(m,a,x)^	4.6 ± 0.5 ^(m,a,x)^	3.5 ± 0.4 ^(n,a,x)^
		1.0%	3.9 ± 0.7 ^(mn,a,x)^	4.4 ± 0.6 ^(m,a,x)^	3.4 ± 0.3 ^(n,a,x)^
	6 mm	0.25%	4.9 ± 1.1 ^(m,a,x)^	4.3 ± 0.9 ^(m,a,x)^	4.3 ± 0.8 ^(m,a,x)^
		0.50%	5.0 ± 1.2 ^(m,a,x)^	4.6 ± 0.7 ^(mn,a,x)^	4.1 ± 0.4 ^(n,a,x)^
		1.0%	4.0 ± 0.9 ^(m,b,x)^	4.0 ± 0.7 ^(m,a,x)^	3.6 ± 0.3 ^(m,a,x)^
		0.25%	4.0 ± 1.1 ^(n,b,y)^	5.1 ± 0.9 ^(m,a,x)^	4.1 ± 0.3 ^(n,a,x)^
	12 mm	0.50%	4.9 ± 1.0 ^(m,a,x)^	4.3 ± 0.5 ^(mn,a,x)^	3.6 ± 0.4 ^(n,a,x)^
		1.0%	3.7 ± 0.7 ^(m,b,x)^	4.3 ± 0.6 ^(m,a,x)^	4.1 ± 0.9 ^(m,a,x)^

Three-way variance analysis was used, comparisons; (xy): for fiber length (ab): for concentration, (mn): for fiber material; values with different superscript letters indicate a significant difference (the first comparisons of triple parameters are colored in same color to emphasize the values to be compared).

## Data Availability

Not applicable.
